# Taste Preference for Salt Predicts Salt Intake in a Chinese Population

**DOI:** 10.3390/nu16132090

**Published:** 2024-06-29

**Authors:** Qingfang He, Xiaofu Du, Lixin Wang, Yujia Fang, Jieming Zhong, Ruying Hu

**Affiliations:** Zhejiang Provincial Center for Disease Control and Prevention, No. 3399 Binsheng Road, Hangzhou 310051, China

**Keywords:** taste preference for salt, urinary sodium and potassium excretion, urinary sodium-to-potassium ratio, hypertension, knowledge, attitude and behavior

## Abstract

Objective: This study describes the association between taste preference for salt and actual salt intake, thus guiding and refining personal and public health campaigns designed to lower salt intake in China. Methods: A cross-sectional survey of 1489 residents aged 18 to 69 years was conducted in 2017 in China. A multistage random sampling strategy was used, and a combination of questionnaires and physical and laboratory measurements were conducted to collect baseline characteristics and knowledge, attitudes, and behavior (KAB) related to salt. A 24 h urine collection was obtained for sodium and potassium excretion analysis. Participants were divided into two groups, light taste preference and salty taste preference, according to their answer to the question “Compared to others, how do you think your taste preference is for salt?”. Results: The mean age of the 1489 participants was 46.26 years, 48.9% were males, over 1/3 (35.7%) were identified as hypertensive, and 317 (21.3%) self-reported a salty taste preference. The mean of 24 h urinary sodium excretion was 167.32 mmol/24 h, corresponding to 9.79 g salt/d intake, and the sodium-to-potassium ratio (Na/K) was 4.90. The 24 h urinary sodium excretion of salty taste preference (177.06 mmol/24 h) was significantly higher than that of light taste preference (164.69 mmol/24 h). The multiple logistic regression analysis showed that the salty taste preference group had significantly higher 24 h urinary sodium (*ORa*(95%*CI*) = 1.004(1.002–1.006)), diastolic blood pressure (DBP), proportion of greasy food preference, and drinking levels, but lower potassium excretion, response levels to most KAB questions, and regular physical activity compared to the light taste preference group. Conclusion: Self-reported taste preference for salt predicted actual salt intake, which was verified by 24 h urinary sodium monitoring. Taste preference for salt could be used as a proxy for intake in terms of targeted salt intake, nutrition, and health education.

## 1. Introduction

According to the Global Burden of Disease (GDB) study, 10.9 million deaths worldwide were attributable to unhealthy diets in 2017. As one of the dietary factors, a diet high in sodium was the leading risk (the ranking rose from 18th in 2007 to 12th in 2017) for mortality, accounting for 3.20 million (1.42–5.45) deaths, among which the key causes of death were high sodium intake resulting in stroke and heart disease [1]. High sodium intake increases the risk for hypertension, and hypertension is an important risk factor for cardiovascular disease [2,3]. In addition, the ratio of sodium to potassium (Na/K) intake is also closely related to hypertension [4]. Sodium intake is a modifiable determinant of hypertension, so reducing dietary salt is one of the most effective approaches for large-scale non-communicable disease (NCD) prevention. Reducing dietary salt has been reported to be associated with a decline hemorrhagic stroke rate in some areas in Japan and Finland, where salt intake was previously more than 20 g per day [5,6]. Through systematic salt reduction strategies, Finland’s per capita daily salt intake fell from about 12 g in 1979 to less than 9 g in 2002 [7]. An approximately 10 mmHg fall in the population average of diastolic blood pressure, and about 60% decrease in deaths from both stroke and ischemic heart disease among 30–59-year-old men and women from 1972 to 1992 [8]. In China, household salt reduction achieved success, with the average personal daily cooking salt consumption at 9.3 g in 2020, a decrease of 1.2 g compared to 2012 and 2.7 g compared to 2002 [9]. However, it is still much higher than the theoretical minimum risk exposure level of diet sodium intake (24 h urinary sodium 1–5 g per day) [1].

Twenty-four-hour urinary sodium excretion is the gold standard for monitoring sodium intake [10,11]. However, 24 h urine collection is difficult and inconvenient. Although some studies showed that self-reported dietary habits were not a reliable way to estimate salt intake [12,13], increasingly more people are paying attention to the association between taste preference for salt and actual salt intake. Drewnowski et al. reported that taste perception and preference were unrelated to sodium intake both in young adults and in older respondents [14]. However, LI Yang et al. recruited 12 subjects with heavy-, medium-, and light-salty taste preference and provided with three-day diets, wherein the daily sodium intake and 24 h urinary sodium levels were measured. A positive correlation was observed between the salty taste preference and sodium intake (*p* < 0.05) [15]. Jiang Mengyang et al. investigated the application value of saltiness perception measured and risk assessment of hypertension by 0.3% NaCl solution as standard salinity in adults [16], while LI Hanqi et al. established a taste dataset of the major categories of Chinese cuisine based on crowdsourced data from Chinese recipe websites and quantitatively studied the relationship between dietary taste and chronic diseases. These results indicated that with the increase in salty taste, the risk of hypertension significantly increases, and the risk of death from hemorrhagic stroke showed an upward trend [17]. Hajeong Lee et al. [18] believed that a self-reported salt-eating habit, not salt taste threshold, predicted actual salt intake. Their study limitation was that 24 h urinary sodium was not collected to monitor the sodium intake. Shen Danyang et al. [19] also believed that the 24 h urinary sodium and sodium/potassium ratio (Na/K) of subjects were positively correlated with self-reported salty taste (*p* < 0.05) through an investigation of 1530 family cooks and members in six regions of China.

In this context, this cross-sectional survey was conducted in Zhejiang Province, China, in 2017 to describe the associations between taste preference for salt and actual salt intake, as well as the associations between taste preference for salt and knowledge, attitudes, and behavior (KAB) of salt and chronic diseases. Such information can guide and refine personal and public health campaigns designed to lower salt intake in China.

## 2. Materials and Methods

### 2.1. Survey Content and Methodology

In 2017 a cross-sectional baseline survey was conducted in Zhejiang Province, China. A total of 3 urban areas and 2 rural areas in eastern, northeastern, central, middle western, and southern Zhejiang Province were selected for the survey, and 18- to 69-year-old subjects without disability and psychiatric disorders and living in the selected areas for at least six months before the survey were randomly selected by a stratified multistage randomized sampling method. The survey was approved by the Ethical Review Committee of Zhejiang Provincial Center for Disease Control and Prevention (no. 2019056), and all the subjects signed written informed consent forms.

A combination of questionnaires, physical measurements, and laboratory tests were used to collect the baseline characteristics including demographic characteristics; history and family history of chronic diseases including hypertension, diabetes, stroke, and cardiovascular disease (CVD); and lifestyle factors including smoking, drinking, dietary structure, taste, greasy food intake, and physical activities, as well as physical measurements such as height, weight, waist circumference (WC), and blood pressure.

Fasting venous blood samples were collected to detect fasting blood glucose (FBG), total cholesterol (TC), triglycerides (TGs), low density lipoprotein cholesterol (LDL-C), and high density lipoprotein cholesterol (HDL-C). Blood samples were detected by KingMed Diagnostics (Hangzhou) Co., Ltd. TC and TGs were detected by the enzymatic method, HDL-C and LDL-C were detected by the direct clearance method, and an Abbott biochemical instrument was used.

Participants’ knowledge, attitudes, and behavior (KAB) related to salt and hypertension were collected. We referred to the World Health Organization/Pan American Health Organization (WHO/PAHO) protocol for population-level sodium assessment and adapted it based on a review of the literature and consultations with public health practitioners and experts [20,21,22]. A total of 19 questions were selected.

A total of 1489 residents completed the baseline survey and returned a complete 24 h urine specimen for urinary sodium and potassium excretion, urinary creatinine, and urinary microalbuminuria analysis. The 24 h urine samples were analyzed by KingMed Diagnostics (Hangzhou, China) Co., Ltd. An ion selective electrode method [7] was used for sodium and potassium analyses (C16000, Abbott Corp., Chicago, IL, USA), and urinary creatinine was measured by the picric acid method, with an immunity transmission turbidity method used to measure urinary microalbuminuria (C501, Roche Cobas Corp., Basel, Switzerland). Urinary excretion was the cross-product of the concentration of the analyte multiplied by the 24 h urine volume.

Methods for sample size calculation; sample selection; and the measurements of height, weight, WC, and blood pressure were as shown in the literature [23,24]. The percentage of body fat was measured with a Omron body fat scales (OMRON, Yangzhou, China), which were accurate to 0.1%.

### 2.2. Definition and Grouping

The standard of hypertension refers to 2018 Chinese guidelines for the management of hypertension [25]: systolic blood pressure (SBP) ≥ 140 mmHg and/or diastolic blood pressure (DBP) ≥ 90 mmHg, and/or self-reported use of antihypertensive medication within two weeks.

Diabetes mellitus is defined as FBG ≥ 7.0 mmol/L and/or approved by a health care provider’s diagnosis or based on medication use [26].

According to the 2016 Chinese guideline for the management of dyslipidemia in adults [27], any of the following is defined as dyslipidemia: total cholesterol (TC) ≥ 5.2 mmol/L, total triglyceride (TG) ≥ 1.70 mmol/L, low-density lipoprotein cholesterol (LDL-C) ≥ 3.4 mmol/L, or high-density lipoprotein cholesterol (HDL-C) < 1.0 mmol/L.

Self-reported CVD history included stroke and coronary heart disease.

Body mass index (BMI) = weight (kg)/(height (m)^2^). According to “Criteria of weight for adults” (WS/T 428-2013) [28], BMI (kg/m^2^) was divided into four groups as <18.5, 18.5–24, 24–28, and ≥28. Central obesity [29]: waist circumference ≥ 90 cm in men and ≥85 cm in women.

Excessive body fat [30]: for men ≥20%, and for women ≥30%.

Current smoking refers to smoking more than 1 cigarette per day consecutively or cumulatively over 6 months.

Drinking is defined as a response of ≥1 time a week in the past year; alcoholic beverages included beer, liquor, red wine, and rice wine.

Self-reported physical activity was ≥150 min per week of moderate intensity or a combination of moderate- and high-intensity exercise, or ≥75 min per week of high-intensity exercise.

Participants were divided into two groups, light taste preference and salty taste preference, according to their answer to the question “Compared to others, how do you think your taste preference is for salt?”. Salty taste preference refers to those who selected the answer “salty taste preference”, which means they love a salt-rich diet; correspondingly, light taste preference refers to those who selected the answer “light taste preference”, which means they favor a lightly salted diet.

Greasy food preference refers to those who responded that they “love greasy foods” to the question “Compared to others, how do you think your taste preference is for greasy foods?”.

Dietary structure was divided into three groups, namely, meat lover, vegetarian diet, and a balanced portion of vegetables and meat, according to their answer to the question “Compared with others, what do you think is the main part of your personal diet: meat (meat, eggs, animal offal, etc.), vegetarian (vegetables, soy products, etc.), or balance portion of vegetables and meat?”.

### 2.3. Quality Control

Before the investigation of this cross-sectional baseline survey, the investigators were trained uniformly, and the investigation and measurement were carried out only after they were qualified. A quality control team was established by both the provincial and county (city) CDCs for quality control and on-site investigation of questionnaires, physical measurement, laboratory tests, information entry and so on.

### 2.4. Statistical Analysis

The continuous variables were described by the means ± SD, while categorical data were presented as frequency (*n*) and percentage (%). ANOVA was used to compare the continuous variables between taste groups. The univariate logistic regression analysis was performed to estimate the odds ratio (*OR*) and 95% confidence interval (*CI*) for the associations with baseline characteristics, as well as the KAB questions between the light taste preference group and salty taste preference group. All the significant baseline characteristics from the ANOVA and univariate regression analysis were controlled in the multivariate logistic regression analyses. Statistical analyses were performed with SPSS for Windows (Version 26, SPSS Inc., Chicago, IL, USA). *p* values < 0.05 were considered statistically significant.

## 3. Results

### 3.1. Baseline Characteristics of Study Participants

As shown in Table 1, the average age of the 1489 participants was 46.26 (14.11) years old, 48.9% were males, 98.7% were Han ethnicity, and 56.3% were from rural areas. The education years for <9 years, 9–12 years, and ≥12 years were 32.6%, 46.1%, and 21.3% respectively. Mean SBP and DBP were 129.67 (19.53) mmHg and 79.90 (10.96) mmHg, respectively, and over 1/3 (35.7%) of participants were classified as hypertensive, with 8.9%, 1.1%, 1.2%, and 34.1% having a history of diabetes mellitus, stroke, coronary heart disease, and dyslipidemia, respectively.

Of the 1489 participants, 1172 (78.7%) self-reported light taste preference, 317 (21.3%) self-reported salty taste preference. Those with a salty taste preference were more likely to be male, a current smoker, a participator in drinking, and a greasy food lover. Participants who self-reported a salty taste preference were significantly older and had higher DBP, WC, and percentage of body fat than those with light taste preference, and those who self-reported as having a light taste preference were significantly more likely to have a higher education level, engage in regular physical activity, and eat a balanced portion of vegetables and meat in their diet than those with a salty taste preference.

### 3.2. Associations between Salty Taste Preference and 24 h Urinary Sodium, Potassium Excretion, and Sodium-to-Potassium Ratio (Na/K)

All 1489 participants returned complete 24 h urine specimens for sodium and potassium, creatinine, and microalbuminuria analysis. As shown in Table 1, the mean (SD) of 24 h urinary sodium excretion was 167.32 (72.22) mmol/24 h, corresponding to 9.79 (4.22) g salt/d, which by far exceeded the WHO recommended maximum level of 5 g salt/d [26]. The mean (SD) of 24 h urinary potassium excretion was 38.02 (17.74) mmol/24 h, corresponding to 1.48 (0.69) g/d, and the Na/k (mmol/mmol) was 4.90 (2.42). More than 88.2% participants’ 24 h urinary sodium ≥ 85.47 mmol/24 h (converted into ≥5 g salt/d).

Table 1 also shows that those with salty taste preference had significantly higher 24 h urinary sodium, percentage of 24 h urinary sodium ≥ 85.47 mmol/24 h (5 g salt/d), Na/K, and sodium/creatinine molar ratio but lower potassium excretion compared to those with light taste preference.

**Figure 1:**
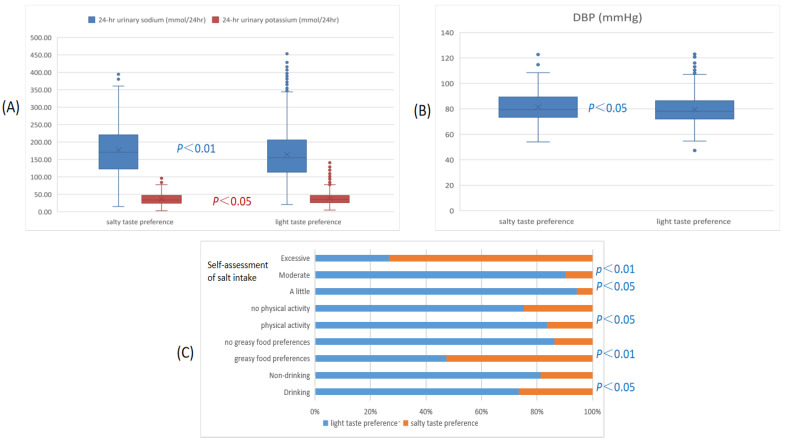
The association between taste preference for salt and self-assessment of salt intake, physical activity, greasy food preferences, drinking, 24 h urinary sodium and potassium excretion, and DBP; (**A**) the association between taste groups and 24 h urinary sodium and potassium excretion; (**B**) the association between taste groups and DBP; (**C**) the association between taste groups and self-assessment of salt intake, physical activity, greasy food preferences, and drinking.

As shown in Table 2 and Figure 1, after adjustment for age, gender, and the significant factors from the ANOVA and univariate regression analysis, we found that those with salty taste preference had significantly higher 24 h urinary sodium, DBP, and percentage of drinking, as well as being more likely to be a greasy food lover, while they had significantly lower levels of potassium excretion and regular physical activity.

### 3.3. Knowledge, Attitude and Behavior (KAB)

As shown in Table 3, more than half of the participants reflected that they had knowledge related to hypertension and the hazards of high-salt diet, but only 39.7% knew that the recommended daily salt intake was less than 6 g salt per day, 32.3% had the knowledge on low-sodium salt, and 23.2% knew that low-sodium salt lowered blood pressure. The four attitude questions were about low-salt diet or labeling sodium/salt content in processed foods, with most participants stating an approval attitude.

As for the behavior questions, 19.9% had self-assessment of excessive salt intake, which was close to 21.3% self-reported salty taste preference. A total of 21.8% participants indicated that they had paid attention to the sodium/salt content label in processed foods, and 15.4% participants actually had used or were using low-sodium salt.

Table 3 also showed that taste groups differed significantly on all but two KAB questions. Overall, light taste preference was linked to significantly higher levels for most KAB questions than salty taste preference. A total of 68.5% of those with salty taste preferences’ self-assessment of salt intake was excessive, 24.6% was moderate, and 6.9% was a little. Those with a light taste preference had a significantly higher percentage of self-assessment of a little salt intake and moderate salt intake, while those with a salty taste preference had a significantly higher percentage of self-assessment of excessive salt intake (Figure 1).

## 4. Discussion

At present, there are several most commonly used methods to assess salt intake: 24 h urinary sodium excretion, spot urinary sodium excretion, dietary recall, salt threshold test, etc. Twenty-four-hour urinary sodium excretion is the gold standard for monitoring sodium intake, and every other method should be verified with it. However, 24 h urine collection is difficult, inconvenient, and cumbersome, particularly for women. Spot urine collection is simple to operate, inexpensive, and can be continuously monitored, but its feasibility and accuracy are still controversial. The dietary survey method is to assess the salt intake of different individuals by investigating their daily meal situation, which mainly relies on the self-reports of different individuals. It is more restricted, time-consuming, laborious, cumbersome, and difficult to operate, as well as being highly subjective. The salt threshold test method is a qualitative method that can be used to determine whether the amount of salt is exceeded but cannot be used to quantify how much salt is eaten. Therefore, it is important to find a relatively simple, inexpensive, and feasible method to assess salt intake.

Taste preference plays a key role in consumer’s food choice and acceptance, which has an important impact on human health. Although taste preference for salt is also a subjective perception, in recent years, a number of studies have supported that self-reported salty taste preference predicts actual salt intake [18,19]. Our aim was to explore the associations between taste preference for salt and socio-demographic characteristics, behavior, lifestyle, chronic diseases, and the KAB of hypertension and salt in a population of 1489 Chinese people. Meanwhile, the relationship between taste preference for salt and 24 h urinary sodium, potassium intake, Na/K, and chronic diseases was examined. 

Our study stated that in the 1489 participants, the mean (SD) 24 h urinary sodium excretion was 167.32 (72.22) mmol/24 h, corresponding to 9.79 (4.22) g salt/d, which was much higher than the WHO recommended maximum salt level of 5 g/d [31], and the Chinese Nutrition Society recommended a maximum salt level of 6 g/d. The 24 h urinary sodium excretion of subjects with salty taste preference (177.06 (70.42) mmol/24 h) was significantly higher than that of subjects with light taste preference (164.69 (72.51) mmol/24 h), confirming that sodium intake was positively correlated with taste preference for salt [15,19]. Furthermore, more than 88.2% participants’ 24 h urinary salt levels were above 5 g, and subjects with salty taste preference had a significantly higher percentage of 24 h urinary sodium ≥ 85.47 mmol/24 h (5 g salt/d), indicating a greater need for salt reduction interventions.

The average age of salty taste preference was significantly older. This might relate to some older dietary habits, when salt was not considered to be unhealthy. Therefore, salt restriction education should start with children [32]. The salty taste perception function was reported to be significantly negatively correlated with age [33], possibly because with the increase in age, the population’s taste sensitivity to salt decreased [34]. This suggested that older adults should be aware that they tended to add more salt to their food to compensate for their low perceptions of salty taste. We would like to suggest them to savor and chew sufficiently during eating to optimize the perception of saltiness. However, Drewnowski et al. studied 24 young adults (aged 20 to 30 years) and 24 healthy older adults (aged 60 to 75 years) and stated that estimated sodium intakes appeared to be independent of taste preference profiles for salty soups, and there was no evidence for an age-related increase in salt taste preference [14]. The difference might be due to the sample size, study methods, and subjects’ sociocultural and socioeconomic status, etc. 

In our study, the proportion of males with salty taste preference was significantly higher than that of females, and this was found in the 24 h urinary sodium (male 173.64 mmol vs. female 161.28 mmol). Laatikainen T et al. reported that, in 2002, the salt intake in 24 h calculated from sodium excretion was about 9.5 g among men in North Karelia and southwestern Finland; among women, the corresponding values were 7.4 g [7]. This might be due to the difference in food preference; body size; and the corresponding total food consumption, energy requirements, and intake between males and females [19], or might be due to females were more sensitive to the salty tastes than males [33], and that females might have controlled their salt intake. Accumulating evidence suggested a role for reproductive hormones in sex differences in NaCl intake and preference; however, there is disagreement in regard to whether the differences are attributable to estrogen [35]. Our survey revealed that after adjustment, the proportion of drinking was significantly higher in salty taste preferences, possibly because dishes that went with drinking were often high oil and high salt in China. Regular physical activity percentage was significantly higher in light taste preferences, possibly because regular physical activity often removed the salt by perspiration.

The mean (SD) 24 h urinary potassium excretion of our study was 38.02 (17.74) mmol/24 h, corresponding to 1.48 (0.69) g/d, which was much lower than the WHO recommended daily potassium intake of 3510 mg [36] and also lower than the average daily potassium intake of Chinese residents from 2011 to 2015 (1827.9 mg) [37]. The 24 h urinary potassium excretion of those with a salty taste preference (35.98 mmol/24 h) was significantly lower than that of those with a light taste preference (38.57 mmol/24 h), indicating that the potassium intake was negatively correlated with taste preference for salt. It was possibly because the proportion of meat lovers and those with greasy food preference in those with a salty taste preference was significantly higher (Table 1), and they resisted vegetables and fruits. Therefore, the intake of potassium-rich foods such as vegetables and fruits was far relatively lower.

The Na/K of our study was 4.90 (2.42), which was much higher than the WHO recommended value of 1.0 [36] and the Dietary Guidelines for Chinese Residents (2016) recommended value of 1.87 [38]. It could be interpreted as more sodium intake and relatively insufficient potassium, resulting in high Na/K, which in those with salty taste preferences was 5.49 (2.52), significantly higher than that of those with light taste preference 4.75 (2.36). DBP (81.43 (11.12) mmHg) was also much higher than those with a light taste preference (79.49 (10.88) mmHg). In 2013, a randomized controlled trial in prehypertensive or hypertensive participants showed that potassium supplementation resulted in a small increase in mean 24 h sodium excretion, and the slight increase in urinary sodium excretion was accompanied by a significant reduction in blood pressure [39], which reduced the effect of sodium intake on blood pressure and damage to target organs such as heart and kidney probably by affecting the expression and phosphorylation of renal Na^+^-K^+^ cotransferase and regulating the kallikrein–kinin system [2,40]. Chu Jie et al. [41] also believed that the increased urinary Na/K was a risk factor for prehypertension, and the urinary Na/K might be more valuable for hypertensive patients’ prognostics.

Our study also found that those with a light taste preference had significantly higher response levels to almost all KAB questions than those with a salty taste preference. Though 68.5% of those with a salty taste preference’s self-assessment of salt intake was excessive, there were still 24.6% with moderate salt level self-assessment, and even 6.9% with little salt level self-assessment. No matter what their actual salt intake was, this indicated that although most salty taste lovers preferred salty foods, some of them had known that too much salt was not good for their heath and consciously controlled the amount of salt to be at a moderate level, or even a little. All these factors also indicated the good feasibility of salt reduction action. Some people could consciously reduce salt intake through salt reduction education. Taste preference for salt could be gradually reduced by long-term adherence to salt restriction. Beauchamp GK et al. [42] also reported that although salt preference has an innate component, optimal salt preference is learned and can be modified by individual experience.

Our study has limitations. Firstly, this is a cross-sectional study, and thus we can describe associations, but no causal relationship can be drawn. Secondly, only a single 24 h urine collection was obtained. A single 24 h urine collection is a measure of sodium intake over the last 1–3 days. The power of a single measurement to predict long-term average concentration is low if the within-person variation is large. Thus, multiple days of 24 h urine collections across time are recommended to assess individual intake measured as a continuous variable or tertiles categories [4]. Thirdly, personal taste preference is not singular, and some participants may have two or more taste preferences. Only taste preference for salt was collected and analyzed in this paper. Lastly, due to limitations of space, there are still many lifestyle and dietary factors not included in this analysis, and statistical adjustment of 2–3 categorical levels is not the best adjustment for the data.

Our study also has several strengths. The 24 h urine collection was obtained to measure sodium and potassium intake and their ratio (Na/K). Assessment of sodium excretion based on 24 h urine collection is considered the gold standard for estimating sodium intake. In our study, the 24 h urine sodium intakes in the salty taste preference group showed significantly higher levels than those with a light taste preference. It turns out that taste preference for salt positively reflected 24 h urine sodium intake and provided a certain reference to evaluate salt intake. Furthermore, our cross-sectional baseline survey was of a large sample size, involving population-based sampling, and was strictly quality controlled with uniformly centralized trained staff and standardized methods.

## 5. Conclusions

In our study, we found that taste preference for salt, although being subjective, positively predicted actual salt intake, which was verified by 24 h urinary sodium monitoring, with this having the potential to provide a relatively simple and some certain reference to evaluate salt intake. The KAB results also support that the attitude of following a low-salt diet can link with the knowledge to reduce dietary salt and engage in sustained behavior. These results support the idea that taste preference for salt could be used in targeted nutrition and health education, salt reduction knowledge training, salt reduction intervention, and initiative salt preference reduction in individual and population dietary counseling and blood pressure management, thus effectively limiting salt intake and developing healthier dietary patterns.

## Figures and Tables

**Table 1:** The univariate logistic regression analysis of baseline characteristics grouped by tastes in participants.

Characteristics	All (*n* = 1489)	Light Taste Preference (*n* = 1172)	Salty Taste Preference (*n* = 317)	*ORc* (95%*CI*)/*p* Value
	*n* (%)			
Age (years) ± SD	46.26 ± 14.11	45.78 ± 14.14	48.04 ± 13.89	0.011 *
<30	260 (17.5)	210 (17.9)	50 (15.8)	1.00
30~40	290 (19.5)	244 (20.8)	46 (14.5)	0.79 (0.51–1.23)
40~50	266 (17.9)	207 (17.7)	59 (18.6)	1.20 (0.78–1.83)
50~60	327 (22.0)	247 (21.1)	80 (25.2)	1.36 (0.91–2.03)
60~70	346 (23.2)	264 (22.5)	82 (25.9)	1.30 (0.88–1.94)
Gender				
Female	761 (51.1)	627 (53.5)	134 (42.3)	1.00
Male	728 (48.9)	545 (46.5)	183 (57.7)	1.57 (1.22–2.02) **
Ethnicity				
Others	20 (1.3)	18 (1.5)	2 (0.6)	1.00
Han	1469 (98.7)	1154 (98.5)	315 (99.4)	2.46 (0.57–10.64)
Urbanity				
Urban	650(43.7)	516(44.0)	134 (42.3)	1.00
Rural	839 (56.3)	656(56.0)	183 (57.7)	1.07 (0.84–1.38)
Hypertension				
Normotensive	957 (64.3)	763 (65.1)	194 (61.2)	1.00
Hypertensive	532 (35.7)	409 (34.9)	123 (38.8)	1.18 (0.92–1.53)
SBP(mmHg) ± SD	129.67 ± 19.53	129.23 ± 19.24	131.31 ± 20.50	0.093
DBP(mmHg) ± SD	79.90 ± 10.96	79.49 ± 10.88	81.43 ± 11.12	0.005 **
Diabetes mellitus				
No	1356 (91.1)	1066 (91.0)	290 (91.5)	1.00
Yes	133 (8.9)	106 (9.0)	27 (8.5)	0.94 (0.60–1.46)
Stroke				
No	1472 (98.9)	1161 (99.1)	311 (98.1)	1.00
Yes	17 (1.1)	11 (0.9)	6 (1.9)	2.04 (0.75–5.55)
Coronary heart disease				
No	1471 (98.8)	1160 (99.0)	311 (98.1)	1.00
Yes	18 (1.2)	12 (1.0)	6 (1.9)	1.86 (0.69–5.01)
Dyslipidemia				
No	981 (65.9)	779 (66.5)	202 (63.7)	1.00
Yes	508 (34.1)	393 (33.5)	115 (36.3)	1.13 (0.87–1.46)
Family history of hypertension				
No	847 (56.9)	668 (57.0)	179 (56.5)	1.00
Yes	642 (43.1)	504 (43.0)	138 (43.5)	1.02 (0.80–1.31)
Family history of diabetes				
No	1260 (84.6)	983 (83.9)	277 (87.4)	1.00
Yes	229 (15.4)	189 (16.1)	40 (12.6)	0.75 (0.52–1.08)
Family history of stroke				
No	1350 (90.7)	1070 (91.3)	280 (88.3)	1.00
Yes	139 (9.3)	102 (8.7)	37 (11.7)	1.39 (0.93–2.06)
Family history of coronary heart disease				
No	1394 (93.6)	1097 (93.6)	297 (93.7)	1.00
Yes	95 (6.4)	75 (6.4)	20 (6.3)	0.98 (0.59–1.64)
Education years				
<9	485 (32.6)	361 (30.8)	124 (39.1)	1.00
9–12	687 (46.1)	553 (47.2)	134 (42.3)	0.70 (0.53–0.93) *
≥12	317 (21.3)	258 (22.0)	59 (18.6)	0.67 (0.47–0.94) *
Current smoking				
No	1154 (77.5)	934 (79.7)	220 (69.4)	1.00
Yes	335 (22.5)	238 (20.3)	97 (30.6)	1.73 (1.31–2.28) **
Drinking				
No	1004 (67.4)	816 (69.6)	188 (59.3)	1.00
Yes	485 (32.6)	356 (30.4)	129 (40.7)	1.57 (1.22–2.03) **
Dietary structure				
Meat lover	137 (9.2)	88 (7.5)	49 (15.5)	1.00
Vegetarian diet	345 (23.2)	281 (24.0)	64 (20.2)	0.41 (0.26–0.64) **
Balanced portion of vegetables and meat	1007 (67.6)	803 (68.5)	204 (64.4)	0.46 (0.31–0.67) **
Greasy food preference				
No	1199 (80.5)	1035 (88.3)	164 (51.7)	1.00
Yes	290 (19.5)	137 (11.7)	153 (48.3)	7.05 (5.31–9.36) **
Physical activity				
No	881 (59.2)	664 (56.7)	217 (68.5)	1.00
Yes	608 (40.8)	508 (43.3)	100 (31.5)	0.60 (0.46–0.78) **
Percentage of body fat(%) ± SD	29.07 ± 6.34	29.12 ± 6.28	28.89 ± 6.56	0.568
Normal	338 (22.7)	284 (24.2)	54 (17.0)	1.00
Abnormal	1151 (77.3)	888 (75.8)	263 (83.0)	1.56 (1.13–2.15) **
WC (cm) ± SD	81.39 ± 9.53	81.16 ± 9.57	82.25 ± 9.34	0.071
Normal	1117 (75.0)	894 (76.3)	223 (70.3)	1.00
Central obesity	372 (25.0)	278 (23.7)	94 (29.7)	1.36 (1.03–1.79) *
BMI (kg/m^2^) ± SD	24.00 ± 3.31	23.94 ± 3.28	24.19 ± 3.38	0.228
<18.5	48 (3.2)	37 (3.2)	11 (3.5)	1.19 (0.59–2.39)
18.5–24	740 (49.7)	592 (50.5)	148 (46.7)	1.00
24–28	531 (35.7)	410 (35.0)	121 (38.2)	1.18 (0.90–1.55)
≥28	170 (11.4)	133 (11.3)	37 (11.7)	1.11 (0.74–1.67)
24 h urinary sodium (mmol/24 h) ± SD	167.32 ± 72.22	164.69 ± 72.51	177.06 ± 70.42	0.007 **
24 h urinary sodium ≥ 85.47 mmol/24 h (converted into ≥5 g salt/d) *^a^*, *n* (%)				
No	175 (11.8)	149 (12.7)	26 (8.2)	1.00
Yes	1314 (88.2)	1023 (87.3)	291 (91.8)	1.63 (1.05–2.52) *
24 h urinary potassium (mmol/24 h) ± SD	38.02 ± 17.74	38.57 ± 18.16	35.98 ± 15.97	0.021 *
24 h urinary sodium-to-potassium ratio (mmol/mmol) ± SD	4.90 ± 2.42	4.75 ± 2.36	5.49 ± 2.52	0.000 **
24 h urinary creatinine(mmol/24 h) ± SD	10.09 ± 4.82	10.18 ± 4.86	9.78 ± 4.69	0.199
24 h urinary sodium/creatinine molar ratio (mmol/mmol) ± SD	19.97 ± 20.24	19.43 ± 21.50	21.97 ± 14.52	0.048 *
24 h urinary potassium/creatinine molar ratio (mmol/mmol) ± SD	4.63 ± 6.33	4.70 ± 6.98	4.34 ± 2.86	0.368
24 h urinary microalbuminuria (mg/24 h) ± SD	12.22 ± 25.74	12.86 ± 27.98	9.84 ± 14.44	0.065
24 h urinary volume (mL/24 h) ± SD	1445.80 ± 442.06	1451.50 ± 437.80	1424.70 ± 457.55	0.338

*ORc:* crude *OR*; *: significance level < 0.05; **: significance level < 0.01. *a*: according to WHO. 2012 Guideline: sodium intake for adults and children, which recommended maximum level of 5 g salt/d. Data are presented as mean ± SD or frequency (*n*) and percentage (%). Samples sizes (*n*), means, and prevalence were unweighted. The percentages are column percents. ANOVA was used to compare the continuous variables between taste groups.

**Table 2:** The multiple logistic regression analysis of baseline characteristics grouped by tastes in participants.

Characteristics	All (*n* = 1489)	Light Taste Preference (*n* = 1172)	Salty Taste Preference (*n* = 317)	*ORa* (95% *CI*)
	*n* (%)			
24 h urinary sodium (mmol/24 h) ± SD	167.32 ± 72.22	164.69 ± 72.51	177.06 ± 70.42	1.004 (1.002–1.006) **
24 h urinary potassium (mmol/24 h) ± SD	38.02 ± 17.74	38.57 ± 18.16	35.98 ± 15.97	0.988 (0.979–0.998) *
DBP (mmHg) ± SD	79.90 ± 10.96	79.49 ± 10.88	81.43 ± 11.12	1.015 (1.002–1.027) *
Drinking	485 (32.6)	356 (30.4)	129 (40.7)	1.379 (1.038–1.831) *
Greasy food preference	290 (19.5)	137 (11.7)	153 (48.3)	6.707 (5.019–8.962) **
Physical activity	608 (40.8)	508 (43.3)	100 (31.5)	0.714 (0.534–0.954) *

*ORa*: adjusted *OR*, adjusted for age (continues), gender (female = 0, male = 1), education years (<9 years = 1, 9–12 years = 2, ≥12 years = 3), DBP (continues), smoking (no = 0, yes = 1), drinking (no = 0, yes = 1), dietary structure (meat lover = 1, vegetarian diet = 2, balance portion of vegetables and meat = 3), greasy food (no = 0, yes = 1), physical activity (no = 0, yes = 1), percentage of body fat (normal = 0, abnormal = 1), WC (normal = 0, central obesity = 1), 24 h urinary sodium (mmol/24 h, continues), 24 h urinary sodium ≥ 85.47 mmol/24 h (no = 0, yes = 1), 24 h urinary potassium (mmol/24 h, continues), 24 h urinary sodium-to-potassium ratio (mmol/mmol, continues), and 24 h urinary sodium/creatinine molar ratio (mmol/mmol, continues), which were the significant factors from the univariate regression analysis. Data are presented as mean ± SD or frequency (*n*) and percentage (%). Samples sizes (*n*), means, and prevalence were unweighted. The percentages are column percentages. *: significance level ˂ 0.05; **: significance level ˂ 0.01.

**Table 3:** The univariate logistic regression analysis of knowledge, attitudes, behavior grouped by tastes.

Characteristics	All (*n* = 1489)	Light Taste Preference (*n* = 1172)	Salty Taste Preference (*n* = 317)	*OR_c_* (95%*CI*)
	*n* (%)			
Knowledge				
Know the diagnostic criteria of hypertension	793 (53.3)	641 (54.7)	152 (47.9)	0.76 (0.60–0.98) *
Know the hazards of hypertension	1120 (75.2)	899 (76.7)	221(69.7)	0.70 (0.53–0.92) *
Know the risk factors of hypertension	1176 (79.0)	946 (80.7)	230 (72.6)	0.63 (0.47–0.84) **
Know salt restriction 6 g per day per person	591 (39.7)	495(42.2)	96 (30.3)	0.59 (0.46–0.78) **
Know low-salt diet helps lower blood pressure	990 (66.5)	806 (68.8)	184 (58.0)	0.63 (0.49–0.81) **
Know the hazards of high-salt diet	1060 (71.2)	858 (73.2)	202 (63.7)	0.64 (0.49–0.84) **
Know who needs a low-salt diet	1178 (79.1)	950 (81.1)	228 (71.9)	0.60 (0.45–0.80) **
Know low-sodium salt	481 (32.3)	401 (34.2)	80 (25.2)	0.65 (0.49–0.86) **
Know low-sodium salt helps control blood pressure	345 (23.2)	290 (24.7)	55 (17.4)	0.64 (0.46–0.88) **
Attitude				
Approve of promoting low-salt diet	1035 (87.6)	1048 (89.4)	257 (81.1)	0.51 (0.36–0.71) **
Approve of low-salt diet	1316 (88.4)	1065 (90.9)	251 (79.2)	0.38 (0.27–0.54) **
Approve of labeling sodium/salt content in processed foods	1102 (74.0)	888 (75.8)	214 (67.5)	0.66 (0.51–0.87) **
Believe that labeling sodium/salt content in processed foods helps to choose low-salt foods	1070 (71.9)	856 (73.0)	214 (67.5)	0.77 (0.59–1.00)
Behavior				
Self-assessment of salt intake				
A little	388 (26.1)	366 (31.2)	22 (6.9)	1.00
Moderate	805 (54.1)	727 (62.0)	78 (24.6)	1.78 (1.09–2.91) *
Excessive	296 (19.9)	79 (6.7)	217 (68.5)	45.70 (27.68–75.45) **
Received publicity or education on low-salt diet	786 (52.8)	632 (53.9)	154 (48.6)	0.81 (0.63–1.04)
Attention to the sodium/salt content label in processed foods	325 (21.8)	280 (23.9)	45 (14.2)	0.53 (0.37–0.74) **
Plan to reduce salt intake	1156 (77.6)	949 (81.0)	207 (65.3)	0.44 (0.34–0.58) **
Take initiative to reduce salt intake	895 (60.1)	764 (65.2)	131 (41.3)	0.38 (0.29–0.48) **
Using or used low-sodium salt	229 (15.4)	193 (16.5)	36 (11.4)	0.65 (0.44–0.95) *

*ORc*: crude *OR*. *: significance level ˂ 0.05; **: significance level ˂ 0.01. Data are presented as frequency (*n*) and percentage (%).

## Data Availability

The data presented in this study are available on request from the corresponding author. The data are not publicly available due to the project in progress and privacy.

## Abbreviations

Na/K: sodium-to-potassium ratio; KAB: knowledge, attitude and behavior; SBP: systolic blood pressure; DBP: diastolic blood pressure; BMI: body mass index; SD: standard deviation; CI: confidence interval; OR: odds ratio. WC: waist circumference; FBG: fasting blood glucose; TC: total cholesterol; TG: triglyceride; LDL-C: low density lipoprotein cholesterol; HDL-C: high density lipoprotein cholesterol; WHO: World Health Organization; PAHO: Pan American Health Organization; CVD: cardiovascular disease; CDC: Center for Disease Control and Prevention.
